# Real World Effectiveness of rTMS in Depression and Anxiety

**DOI:** 10.1192/bjo.2024.257

**Published:** 2024-08-01

**Authors:** Milind Thanki, Paul Briley, Sarah Ottahal, Sudheer Lankappa, Mohammad Zia Ul Haq Katshu

**Affiliations:** 1 Nottinghamshire Healthcare NHS Foundation Trust, Nottingham, United Kingdom; 2 Sheffield Hallam University, Sheffield, United Kingdom; 3 Institute of Mental Health, University of Nottingham, Nottingham, United Kingdom

## Abstract

**Aims:**

Repetitive Transcranial Magnetic Stimulation (rTMS) is a non-invasive brain stimulation recommended by NICE for treatment of depression with minimal side-effects and a high patient acceptability. Our aim was to assess the effectiveness of rTMS in real world clinical service in alleviating symptoms of depression and anxiety.

**Methods:**

All patients receiving rTMS in our Centre for Neuromodulation Services (CNS) received 5 daily treatment sessions a week for a period of 5 weeks (25 sessions in total). All patients routinely completed PHQ-9, BDI-II and GAD-7 measures before and after the course of treatment. The scores on these measures were retrospectively analysed using paired-sample t-test.

**Results:**

All 15 patients completed the PHQ-9 and GAD-7 scales while 10 patients completed BDI-II. Eleven patients (73%) had improved PHQ-9 scores post-treatment with average improvement of 5.5 points which was statistically significant [paired-sample t-test: t(14) = 3.019, p = 0.009]. Nine patients (90%) had improved BDI-II scores post-treatment with average reduction of 36% from baseline which was statistically significant [t(9) = 3.681, p = 0.005]. Eleven patients (73%) had improved GAD-7 scores post-treatment, with average reduction of 4 points. This reduction was also statistically significant [t(14) = 3.038, p = 0.009]. Improvement in all measures was also of a level that would be considered clinically significant for these measures. All patients tolerated the treatment well with no patients dropping out due to side effects.

**Conclusion:**

With the limitation of relatively small sample size, our initial analysis indicates that rTMS treatment offered in real world clinical service is effective in treating symptoms of depression. Although our protocol was not intended to treat anxiety, our patients had remarkable improvement in anxiety symptoms as well.

